# Experimental comparison of the genetic component of pollinator effectiveness in a shrub pollinated by birds, non-flying mammals and European honeybees

**DOI:** 10.1007/s00442-025-05736-x

**Published:** 2025-06-06

**Authors:** Stanislaw K. Wawrzyczek, Siegfried L. Krauss, Susan E. Hoebee, Ryan D. Phillips

**Affiliations:** 1 Department of Ecological, Plant and Animal Sciences, La Trobe University, Melbourne, VIC 3086 Australia; 2 Department of Biodiversity, Conservation and Attractions, Kings Park Science, Fraser Avenue, Kings Park, WA 6005 Australia; 3 School of Biological Sciences, University of Western Australia, Crawley, WA 6009 Australia; 4 Royal Botanic Gardens Victoria, Melbourne, VIC 3004 Australia

**Keywords:** Heterozygosity-fitness correlation, Inbreeding depression, Mating system, Polyandry, *Tarsipes rostratus*

## Abstract

The range and frequency of pollinator movement, influences patterns of pollen dispersal with consequences for plant mating. It is predicted that pollination by birds promotes outcrossing and multiple paternity. Alternatively, pollination by insects or non-flying mammals (NFMs) is expected to increase inbreeding and correlation of paternity, leading to less genetically diverse and less vigorous seedlings. We tested these predictions in *Banksia catoglypta* (Proteaceae)*—*an obligately outcrossing heathland shrub pollinated by honeyeater birds, NFMs (honey possums and rodents) and insects (mostly introduced *Apis mellifera*). Previous research employing selective pollinator exclusion determined that pollination by NFMs was associated with reduced fruit set, compared to flying pollinators. Here, we used microsatellite genotyping of seedling families and a common garden experiment to compare the effectiveness of bird, NFM, and insect pollinators in terms of the genetic quality and vigour of the resulting seedlings. Despite floral traits of *B. catoglypta* suggesting adaptation to mammalian pollinators, pollination solely by NFMs led to lower multilocus outcrossing rates (*t*m, indicating biparental inbreeding) and higher correlation of paternity (*r*p), compared to pollination by flying pollinators (birds and insects combined). Otherwise, there were no significant differences between pollination treatments and open-pollinated controls. Overall, seedling survival was positively correlated with individual heterozygosity, with seedlings resulting from pollination solely by NFMs least likely to survive, and exclusion of NFMs associated with increased seedling survival*—*although these effects were slight. Our results highlight the importance of quantifying plant mating and seedling fitness for understanding the consequences of pollination by different animals.

**Supplementary Information:**

The online version contains supplementary material available at 10.1007/s00442-025-05736-x.

## Introduction

For most plants, outcross pollination is advantageous through minimizing expression of deleterious alleles and enhancing offspring fitness (Barrett 1998; Charlesworth and Willis 2009; Roberts et al. 2014; Barrett and Harder 2017). Moreover, a diversity of pollen sires (i.e. polyandry) can increase plants’ female fitness by providing greater opportunity for selection among competing paternal genotypes or advantageous gene combinations, resulting in more vigorous offspring (Burd 1998; Krauss 2000; Winsor et al. 2000; Keller and Waller 2002; Heliyanto et al. 2005; Breed et al. 2012, 2014). Alternatively, self-pollination and biparental inbreeding tend to be associated with reduced offspring fitness (Vaughton and Ramsey 2006; Yates et al. 2007; Charlesworth and Willis 2009; Llorens et al. 2013), while low paternal diversity can be detrimental to overall progeny performance (Breed et al. 2012; Nora et al. 2016).

For many flowering plants, their reproductive success depends on animal pollinators moving pollen between flowers (Mitchell et al. 2009; Barrett and Harder 2017). However, pollinators are predicted to forage for nectar or pollen by preferentially moving to nearby flowers and between plants growing in the same area to maximize energy intake relative to expenditure (Pyke et al. 2021). Such a foraging strategy would result in a pattern of pollen dispersal dominated by short-distance pollen movements. Such leptokurtic dispersal of pollen may lead to biparental inbreeding if nearby plants are closely related, which is particularly likely to arise in species lacking adaptations for long distance dispersal by seeds (Vekemans and Hardy 2004).

The expectation that pollinators tend to visit multiple flowers within a plant and move mostly between neighbouring plants broadly matches empirical observations of foraging behaviour of individual Honeybees (*Apis mellifera*) (Ribbands 1949; Butz Huryn 1997; Akter et al. 2017) and nectar-feeding birds (Hopper and Burbidge 1978; Pyke 1981; Collins et al. 1990; Evans and Bunce 2000). However, in the case of birds, foraging at flowers is often disrupted by intra- and inter-specific aggression (McFarland 1986; Armstrong 1991; Camfield 2006; Phillips et al. 2014; Rodríguez-Flores and Arizmendi Arriaga 2016; Ritchie et al. 2021) and intermittent bouts of foraging for insect prey (Halse 1978; Pyke 1980; McFarland 1986). Additionally, limited pollen grooming by birds results in high rates of pollen carry-over between foraging bouts (Minnaar et al. 2018). Consequently, pollination by birds should generally be advantageous for plants through promotion of outcrossing (Krauss et al. 2009, 2017; Whelan et al. 2009; Wessinger 2021) and paternal diversity (Hoebee 2002; Breed et al. 2015; Bezemer et al. 2019). However, some bird-pollinated plants may experience low paternal diversity (correlated paternity) due to interspecific competition for pollinators (Kestel et al. 2021). In contrast to pollination by birds, pollination by some insects may result in elevated rates of selfing and biparental inbreeding as a consequence of moving pollen mostly between flowers on the same plant (geitonogamy), shorter interplant movements and, in the case of bees, more frequent grooming of pollen collected on their bodies reducing pollen carry-over (Johnson et al. 2001; Johnson and Pauw 2014; Diller et al. 2022). However, this is not likely to be the case for all plants and all insects due to differences in the plants’ flowering intensity and breeding systems, and the foraging behaviour among pollinating insects. For example, in *Protea caffra,* pollination by birds and beetles resulted in equivalent outcrossing rates, which was attributed to the long inter-plant movements of the beetles (Steenhuisen et al. 2012), while Valverde et al. (2019) showed that genetic diversity of pollen transferred can vary among functional groups of insect pollinators.

The genetic consequences of pollination by non-flying mammals (NFMs) for plant mating are unknown. Based on behavioural studies, NFMs may be more effective than birds and insects at transferring pollen on a per-visit basis (Carthew 1993). At least in brush-type flowers they tend to contact the flowers with much larger area of their bodies (Goldingay et al. 1987; Carthew 1993), and pollen adheres to fur more strongly than to feathers (Muchhala and Thomson 2010). However, compared to nectarivorous birds, NFMs are predicted to make shorter inter-plant movements (Evans and Bunce 2000), while the amount of pollen they transfer between the flowers may be reduced because of grooming between foraging bouts (Johnson and Pauw 2014). For key pollinating groups of NFMs such as rodents and the marsupial honey possum, empirical documentation of movements during bouts of foraging for nectar and pollen is scant, though honey possums are typically observed feeding on multiple inflorescences per plant (R. Phillips, personal observation). Nonetheless, some NFMs may move between plants more often than expected, despite a low incidence of aggressive interactions. For example, Carthew (1994) used fine thread to track movements of two scansorial marsupials and one glider among flowering plants of three *Banksia s*pecies and found that they mostly moved between inflorescences on different plants that were at least 5 m apart, despite the availability of many inflorescences per plant. Similarly, Evans and Bunce (2000) found that Eastern Pygmy-possums (*Cercartetus nanus*) foraging on *Banksia integrifolia* visited fewer inflorescences per tree and covered only slightly shorter distances during inter-plant movements compared to honeyeater birds (Meliphagidae). Given that the NFM species in these studies were not obligate nectarivores, their foraging movements may partly reflect the need to find diverse food items beyond pollen and nectar (Van Tets and Whelan 1997). Thus, past studies generate conflicting expectations regarding the consequences of NFM pollinators’ foraging behaviour for plant mating.

The Australian plant genus *Banksia* L.f. (Proteaceae) is characterized by large inflorescences that are an important source of nectar and pollen for diverse assemblages of pollinators including birds, NFMs and insects (Paton and Turner 1985; Collins and Rebelo 1987; Lamont and Collins 1988; Ramsey 1988; Carthew 1993; Wawrzyczek et al. 2024a). Evidence from pollen loads, observation of animals foraging on the flowers and selective pollinator exclusion experiments, suggest that the relative importance of these different functional pollinator groups can vary among *Banksia* species: some are primarily pollinated by birds (Ramsey 1988) or a mix of birds and NFMs (Goldingay et al. 1987; Cunningham 1991; Carthew 1993; Wooller and Wooller 2003; Wawrzyczek et al. 2024a). In some species, native insects may also contribute to pollination (Lamont and Collins 1988; Ramsey 1988), although the introduced *A. mellifera* is often the most frequent visitor and an effective pollinator of *Banksia* flowers (Vaughton 1992; Wooller and Wooller 2001; Gilpin et al. 2017; Wawrzyczek et al. 2024a). This diversity of pollen vectors makes *Banksia* a powerful study system for experimentally comparing the consequences of different pollinator functional groups for plant mating.

Among the banksias, the heathland shrub *B. catoglypta* is a particularly suitable model for pollination studies. Endemic to southwest Western Australia, this species flowers profusely and reliably produces large numbers of fruit, while its low shrub form facilitates experimental manipulation of flowers and monitoring of floral visitors. It is obligately outcrossing and effectively pollinated by honeyeaters, NFMs (including honey possums and mice) and the introduced *A. mellifera* (Wawrzyczek et al. 2024a). An earlier study used a field-based selective pollinator exclusion experiment to determine the contribution of these functionally different pollinator groups to fruit set of *B. catoglypta* (Wawrzyczek et al. 2024a). Here, we genotyped the maternal plants and seedlings resulting from that experiment to compare the consequences for plant mating of pollination by birds, NFMs, and *A. mellifera*. We then used a common garden experiment to determine whether seedling vigour was affected by pollinator functional group and tested for a correlation between seedling vigour and individual heterozygosity to determine whether any effects due to selective exclusion of pollinators had a genetic basis. We hypothesized that (1) flying pollinators (birds and insects) will contribute more genetically diverse outcross pollen than NFMs, resulting in higher seedling heterozygosity, while pollination by NFMs will lead to increased inbreeding and correlated paternity, and reduced seedling heterozygosity and vigour. Further, we hypothesized that (2) these effects will be attributable to pollination by birds rather than insects. Finally, we expected that (3) seedling growth and survival will be positively correlated with individual heterozygosity.

## Methods

### Study species

*Banksia catoglypta* is a narrow range endemic occurring in the species-rich heathlands (kwongan) of the Lesueur Sandplain region in Western Australia (see Wawrzyczek et al. 2024a for full details). It can grow at relatively high densities (typically, 4–10 adult plants in a 200 m^2^ plot; SKW, unpublished data) although its distribution within the landscape can be somewhat patchy. We undertook our study at Hi Vallee Farm in Western Australia (30°07′13ʺ S, 115°24′15ʺ E), one of only two confirmed extant populations of *B. catoglypta*.

*Banksia catoglypta* is a low shrub that produces large gold-yellow inflorescences of 85–110 rigid flowers with secondary pollen presentation on the tip of the style (pollen presenter) ca. 4.5 cm away from the nectaries located at the base of the florets. The long distance between stigma and nectary suggests adaptation to pollination by birds. However, as is common for kwongan-endemic banksias, the flowers of *B. catoglypta* are somewhat concealed among foliage and strongly scented—traits that likely facilitate pollination by NFMs (Wawrzyczek et al. 2024b). The experimental pollination has indicated that the species is obligately outcrossing, with open-pollinated inflorescences typically producing 2–6 woody follicles each containing up to two seeds (Wawrzyczek et al. 2024a). Like most banksias, the seeds feature a papery wing that facilitates dispersal by wind (Orchard et al. 1999). However, *Banksia* seeds are large and heavy, meaning that long distance dispersal (i.e. > 200 m) is likely a rare occurrence (He et al. 2009) and most seeds disperse over much shorter distances (< 10 m; Krauss et al. 2009).

### Pollinator surveys

Concurrently with the experiments presented here, visitation by vertebrate pollinators to *B. catoglypta* flowers was quantified through a total of 2363 hours of surveys using heat and motion-triggered camera traps (see Wawrzyczek et al. 2024a). This work revealed that birds, particularly white-cheeked honeyeater, *Phylidonyris niger*, were the most frequent vertebrate pollinator visiting *B. catoglypta* flowers (0.13 visits per hour; Wawrzyczek et al. 2024a). However, NFMs, including honey possums and rodents, also visited the flowers (0.04 visits per hour; Wawrzyczek et al. 2024a). The majority of the rodents were likely house mice, *Mus musculus*, although some may have been the native ash-grey mice, *Pseudomys albocinereus* (Wawrzyczek et al. 2024a). In addition, surveys of invertebrate pollinators employing direct observation and video recordings revealed that on warm, sunny days, European honeybees, *Apis mellifera*, were very frequent visitors to *B. catoglypta* flowers (median 5 visits per 30 min survey; Wawrzyczek et al. 2024a) while visitation by native insects was negligible due to winter flowering (Wawrzyczek et al. 2024a). The native insects occasionally observed on *B. catoglypta* flowers comprised blowflies (Calliphoridae), small wasps (Thynnidae, Scoliidae) and metallic beetles (Cleridae)—none of which could be considered an effective pollinator as they were unlikely to move appreciable amounts of pollen between the flowers due to infrequent contact with pollen presenters and/or small body size (Wawrzyczek et al. 2024a).

### Selective pollinator exclusion experiment

The present study used *B. catoglypta* seeds arising from the selective pollinator exclusion experiment, which aimed to compare the effectiveness of different functional groups of pollinators in terms of their contribution to fruit set (see Wawrzyczek et al. 2024a for full details). To recapitulate, the following pollination treatments/controls were applied either to entire plants or large flowering branches, replicated in a minimum of seven plants (3–12 inflorescences per plant): (1) ‘Open’ pollination (natural pollination control): inflorescences were marked at the beginning of anthesis and left unmanipulated. (2) ‘Mammals only’ pollination: Flying pollinators were excluded with custom-made fine-mesh (1 mm) bags, which were secured such that a gap of about 3 cm was left at the bottom to allow small mammals to move freely in and out. (3) ‘Birds and insects’ pollination: NFMs excluded by a 15 cm tall drift fences were erected around individual *B. catoglypta* plants. A distance of ca. 20 cm between the nearby shrubs and experimental plants was ensured to prevent NFMs from climbing over the fences using overhanging vegetation. (4) ‘Insects only’ pollination: All vertebrates were excluded by a combination of a plastic drift fence and a semi-rigid, coarse lattice cage (25 × 25 mm mesh) covering either an entire plant or a large flowering branch (see images in Wawrzyczek et al. 2024a: online supplementary material). (5) ‘Pollen supplementation’: open-pollinated inflorescences were hand-pollinated at least twice during anthesis using pollen sourced from at least two other plants growing more than 10 m away. The pollen supplementation treatment was included here to serve as a benchmark for what we expected to be the minimum pollen dispersal distance that should result in mating between unrelated individuals. This expectation was based on the average plant density at the study site, where there were typically at least ten other *B. catoglypta* plants within the 10 m radius of each experimental plant, with studies of other *Banksia * species indicating absence of spatial genetic structure beyond ten meters distance (Krauss et al. 2009).

The efficacy of the exclusions was confirmed through camera trap monitoring and direct observation (see Wawrzyczek et al. 2024a for full details). Because of the low shrub form of *B. catoglypta* entire plants needed to be fenced off to exclude NFMs. This meant that the ‘Mammals only’ treatment and the ‘Open’ control could not be placed on the same plants as the ‘Birds and Insects’ and ‘Insects only’ treatments. Apart from that, we strove to replicate treatments within individual plants to reduce the influence of the potential confounding factors relating to plant position and size. We also ensured all treatments were replicated on plants of similar size and growing no more than several meters apart.

### Common garden experiment

In July 2022, 427 seeds resulting from the pollination treatments and controls described above, were germinated and grown in individual stock tubes in a shadehouse. Up to 12 seeds per maternal plant from a minimum of 7 maternal plants per treatment (Table 1) were released from the follicles by heating them in an oven for 15 min at 60 °C. As much as possible, we used seed from different infructescences and only one of the two seeds from each follicle to minimize the effects of potential correlation of paternity within infructescence/fruit.

**Table 1:** Summary of pollination treatments and replication in the common garden experiment. A total of 23 maternal plants were used with pollination treatments and controls replicated within the same plant whenever possible

Treatment	*N* plants	*N* seeds
Pollen supplementation	7	69
Open	9	104
Mammals only	8	93
Birds & insects	7	76
Insects only	8	85

On 12-Jul-2022, the seeds were sown directly into individual nursery stock tubes filled with 1-part low phosphorous native potting mix (Richgro, Jandakot) and 2-parts (by volume) washed sand to resemble the soil conditions the seedlings would experience in the wild. The stock tubes with partially buried seeds were placed on an elevated bench in a shadehouse at Kings Park Biodiversity Conservation Centre, Perth, Western Australia. They were watered once daily and rotated every 2 weeks to ensure uniform conditions (ambient temperature was not controlled). After 7 weeks, 276 (65%) seeds germinated and had fully extended their cotyledons. At this stage, we moved the plants to an elevated bench under a shade cloth in an outdoor courtyard. We continued once-daily watering and repositioned the plants every 2 weeks. By 29-Sept-2022, six additional seeds had germinated, and the young seedlings had their first true leaves. At this stage, the cotyledon tissue was harvested for DNA extraction from a total of 282 seedlings. In total, 35% of seeds failed to germinate (were either not viable or remained dormant).

**Fig. 1:**
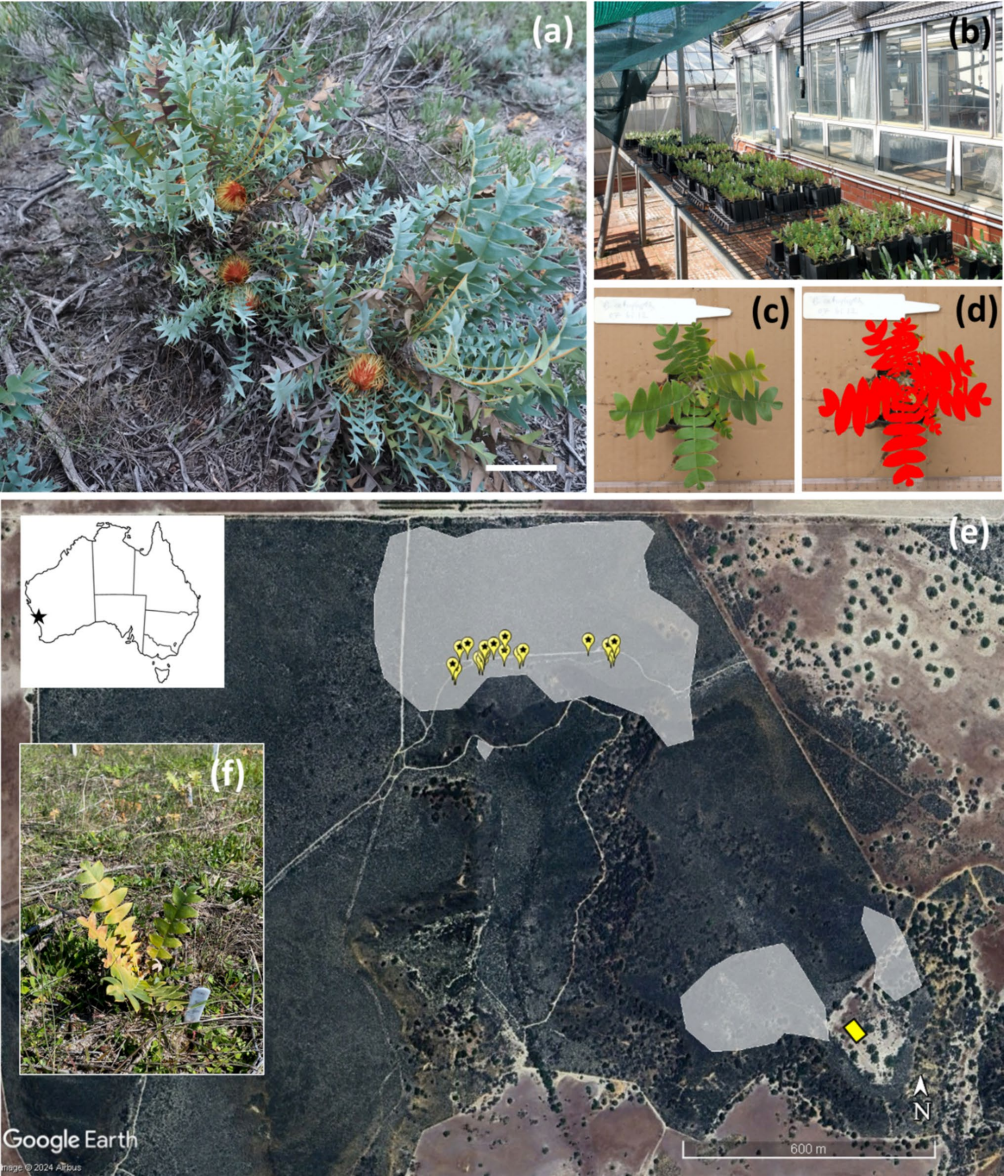
**a** *Banksia catoglypta* with inflorescences in early anthesis, scale bar = 10 cm; **b,** *B. catoglypta* seedlings at Kings Park and Botanic Gardens, Perth, in January 2023, 6 months after sowing; **c**, a seedling photographed in April 2023, and **d** projected area of green leaf captured by ImageJ colour threshold (red highlight) used to compare seedling growth; **e** map of the study site at Hi Vallee Farm, indicating the distribution of *B. catoglypta* (grey polygons, in 2022–2023, a total of 2280 plants were counted in the combined area shown), maternal plants from which seed were collected following the selective pollinator exclusion experiment (yellow markers), and the site where experimental seedlings were planted (yellow rectangle); **f** one of the *B. catoglypta* seedlings planted at the site, with yellowing of leaves (photographed three weeks after planting)

Late in December 2022, during a severe heatwave, some seedlings died apparently due to being in the blind spot of the watering system. By 31-Mar-2023, nine months after sowing, 248 seedlings remained alive (Fig. 1b). Because most of the seedlings were yellowing around the leaf margins, we applied a small amount of natural seaweed-based fertilizer (50 mL of Seasol® Complete Garden Health Treatment diluted in 10 L of water, distributed over all seedlings).

At this time, we estimated the amount of plant growth to compare among the pollination treatments. This was done by taking digital photographs of the seedlings, positioning a camera on a tripod at a height of 1.2 m directly above the plants with plain brown cardboard for background. The seedlings’ label and a ruler for scale were placed in each photo. We then used ImageJ (version 1.53; http://imagej.nih.gov/ij) to measure projected green leaf area using the colour threshold function. We set the thresholds at: Hue 40–110, Saturation 50–255, and Brightness 50–255, which consistently excluded all dead (brown) leaf tissue from the measurements and mostly excluded very young shoots (Fig. 1c,d). The measurements were repeated in July 2023, 12 months after sowing. Because the two measurements were strongly correlated (*R*^2^ = 0.86, *p < *0.001), only the latter is reported.

On 3-Aug-2023 the seedlings were planted in a small area adjacent to the study population at Hi Vallee Farm in Western Australia (Fig. 1e; 30°07′13″ S, 115°24′15″ E; Wawrzyczek et al. 2024a). This area was cleared of native vegetation in 1985 but it was not farmed. The seedlings were planted 1 m apart in a 24 m × 11 m grid in random order and received ca. 20 mm of rain in the evening following the planting. The site was inspected 3 weeks after planting. At this time, most plants were alive, but many had yellowing leaves (Fig. 1f). The seedlings’ survival was scored on 10-Oct-2023, 15 months after germination and 3 months after transplanting the seedlings. Plants with at least one mostly green leaf were scored as ‘alive’ (1) and otherwise as ‘dead’ (0). The green leaf area measurements and survival in the field data were obtained for 234 and 227 seedlings, respectively. The remaining seedlings had either died during the summer heatwave, could not be identified due to label fading, or were apparently browsed after planting.

### DNA extraction and genotyping

The genomic DNA was extracted from dried leaf tissue of maternal plants using Plant DNeasy Kit (QIAGEN) and from fresh cotyledon tissue of seedlings using the CTAB method (adapted from Doyle 1990). All DNA samples were eluted and resuspended with H_2_O adjusting the concentration of extracts to approximately 20–100 ng µL^−1^.

Twenty polymorphic microsatellite markers were specifically developed for *B. catoglypta* by the Australian Genome Research Facility (AGRF). To genotype the plants, we used eight of these markers (Table S1) in two multiplex PCR reactions employing the three primer method of Blacket et al. (2012). The reactions were performed in-house at La Trobe University using Type-it Microsatellites kit (QIAGEN) (see supplementary material 1 for primer sequences and details of PCR protocol). Amplified DNA samples were then sent for capillary electrophoresis at AGRF. The returned raw electropherogram files were then analyzed in-house using Geneious Prime (version 2023.1.1) to manually call amplicons (microsatellite alleles). The resulting data set was checked for consistency of amplicons within seed families. We inferred maternal genotype from the progeny arrays in 19 instances of null homozygous loci (or failed amplification), and 12 cases of apparent null heterozygous loci. In a single ambiguous case, found at one locus (Bca10), the most frequent allele across the data set was inserted as the most likely maternal allele. The full data set (maternals and seedlings) was checked for additional null alleles using Micro-Checker (version 2.2.3), which indicated (with 95% confidence after 1000 permutations) only slight excess of homozygotes in one locus (Bca06, 142 observed vs. 120 predicted). This was considered unlikely to affect the results as the small number of additional null alleles at this locus should affect all pollination treatments equally.

### Mating system data and statistical analyses

To assess the effect of pollination by different pollinator functional groups on the mating system of *B. catoglypta*, we first used GenAlEx (Peakall and Smouse 2006, 2012) to estimate standard population genetic parameters for the adult population and seedling cohorts grouped by pollination treatment (five groups). Next, we used MLTR (Ritland 2002) to estimate the following family level parameters of the mating system: multi-locus and single locus outcrossing rates (*t*m and *t*s, respectively), correlation of paternity (*r*p), and correlation of selfing (*r*s). Derived from these estimates, 1/*r*p can be interpreted as effective number of pollen donors (*N*ep). Further, 1 − *r*s approximates the fraction of apparent (effective) selfing due to biparental inbreeding (Ritland 2002). Alternatively, *t*m – *t*s is commonly used to estimate biparental inbreeding. However, *t*m – *t*s tends to underestimate biparental inbreeding and is only informative if true (uniparental) selfing also occurs (Ritland 2002). Here, we expect no self-fertilization based on the results of Wawrzyczek et al. (2024a) whereby a single seed was produced following geitonogamous hand pollination of 13 inflorescences. This seed germinated and the seedling was genotyped (using the same eight microsatellite loci used in this study), which showed that it was in fact outcrossed. In the absence of true selfing, all apparent selfing is due to biparental inbreeding and 1 − *r*s should be close to 1, and *t*m – *t*s close to zero. Again﻿, ﻿we g﻿rouped the experimental seedlings by pollination treatment and specified known maternal genotypes. Whenever multiple treatments were applied to the same maternal plant, we repeated the maternal genotype for groups of seedlings in different treatments. We used the expectation maximization method and calculated the 95% CI’s for the estimates by bootstrapping entire families 1000 times. Finally,﻿ to assess ﻿whether there was a genetic component underlying the variation in seedling growth and survival after planting in the field, we conducted an individual-level heterozygosity-fitness correlation test (Szulkin et al. 2010). For each maternal plant and seedling, we used Microsoft Excel to calculate individual heterozygosity (*H*i) expressed as the number of heterozygous microsatellite loci (an integer ranging from 0 to 8). We then fitted generalized linear mixed models (GLMMs) to test for correlation between *H*i and seedling growth and survival.

To test our hypotheses, we considered comparisons among the selective pollinator exclusion treatments and between each of these treatments and open-pollinated controls. Additionally, we tested for the effect of outcross pollen supplementation relative to open-pollinated control and for differences in individual heterozygosity between experimental seedlings and maternal plants. We used the R-packages ‘glmmTMB’ (Brooks et al. 2017) to fit GLMMs, and ‘emmeans’ (Lenth 2024) for pairwise tests. We fitted a Poisson model with log-link function for seedling heterozygosity, Gaussian distribution for the leaf area, and binomial distribution for seed germination and seedling survival. In all models, we specified maternal plant as a random effect (Zuur et al. 2009). Data figures were created using R-packages tidyverse/ggplot2 (Wickham et al. 2019). All analyses were conducted in R version 4.2.3 (R Core Team 2023). We report full *P*-values and use language of evidence (Muff et al. 2021), instead of referring to the conventional significance threshold at α = 0.05. This approach emphasizes that *P-*values are a continuous measure of statistical support. However, for consistency, we consider* P*-values < 0.001 as very strong evidence, < 0.01 strong, < 0.05 moderate, < 0.10 weak, < 0.20 little to no evidence, and > 0.20 no evidence. Alternatively, we consider a comparison statistically significant if the 95% CI’s of the estimates of difference do not overlap zero.

## Results

### Microsatellite data and genetic diversity

The eight microsatellites used to genotype *B. catoglypta* plants showed relatively low polymorphism, with between 2 and 5 alleles per locus (26 in total, Table S1). In total, 22 maternal plants and 249 seedlings (including some that died during the summer heatwave) were successfully genotyped. One family was discarded because the maternal genotype was missing data at six microsatellite loci and, with only three seedlings in the family, it could not be reliably inferred from progeny arrays. We permitted missing data for one locus in 10 seedlings and for two loci in a further three seedlings. These loci were treated as null allele homozygous in the following analyses.

There was considerable variation in the population genetic parameters estimated in GenAlEx (Peakall and Smouse 2006, 2012) for the maternal plants and seedling cohorts. The adult plants were overall more heterozygous (*H*o) than the offspring arising from open pollination. The seedlings in the ‘Mammals only’ treatment were the least heterozygous cohort, while those in the ‘Birds & insects’ treatment were the most heterozygous (Table 2). Expected heterozygosity (*H*e) and unbiased expected heterozygosity (u*H*e) were highest in the ‘Birds & insects’ treatment (higher than in the maternal plants and the ‘Open’ control)—and lowest in the ‘Mammals only’ treatment (Table 2). Pollen supplementation of open-pollinated inflorescences did not have an appreciable effect on any of the parameters. The effect of pollination treatments on the coefficient of inbreeding (*F*) were negligible as *F* was very low in all groups (Table 2).

**Table 2:** Population genetic parameters estimated in GenAlEx (Peakall and Smouse 2006, 2012) based on genotypic data for eight microsatellite loci for maternal plants pools of seedlings grouped by pollination treatment, mean (± SE)

	*N*	*H*o	*H*e	u*H*e	*F*
Maternal	22	0.563 (± 0.073)	0.475 (± 0.040)	0.487 (± 0.041)	− 0.157 (± 0.091)
Pollen suppl	49 (7)	0.477 (± 0.043)	0.446 (± 0.035)	0.450 (± 0.035)	− 0.064 (± 0.032)
Open	59 (9)	0.457 (± 0.048)	0.455 (± 0.046)	0.459 (± 0.046)	− 0.022 (± 0.055)
Mammals only	47 (9)	0.414 (± 0.047)	0.423 (± 0.052)	0.427 (± 0.053)	− 0.002 (± 0.043)
Birds & insects	44 (7)	0.522 (± 0.039)	0.507 (± 0.025)	0.513 (± 0.025)	− 0.023 (± 0.038)
Insects only	50 (8)	0.454 (± 0.042)	0.461 (± 0.028)	0.466 (± 0.029)	0.012 (± 0.072)

*N* number of individuals genotyped (seedling families); *H*o observed heterozygosity; *H*e expected heterozygosity; u*H*e unbiased expected heterozygosity; *F* coefficient of inbreeding

**Fig. 2:**
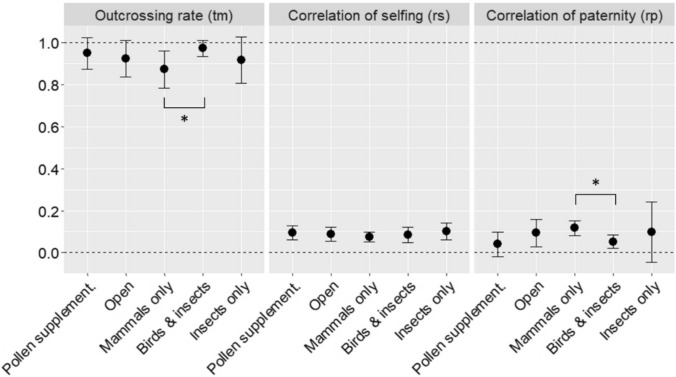
Comparison of the MLTR (Ritland 2002) estimates of the mating system parameters among seedling families grouped by pollination treatments. Error bars = 95% CI based on 1000 bootstraps resampling seedling families. The asterisks indicate significant pairwise differences between pollination treatments at α = 0.05 based on 1000 bootstraps of estimate differences

The mating system parameters estimated by MLTR indicated high multilocus outcrossing (*t*m) rates, ranging from 0.87 to 0.97 and differing significantly from 1 only for the ‘Mammals only’ treatment (Fig. 2). The very low values of correlation of selfing (*r*s), and consequently very high values of 1 − *r*s for all treatments indicated that no, or very little, uniparental inbreeding had occurred (Fig. 2). Therefore, all apparent selfing should be attributed to biparental inbreeding (Ritland 2002). Consistent with this result, the values of *t*m – *t*s were very low for all treatments, ranging from − 0.01 to 0.06. Evidence of biparental inbreeding was detected only for the ‘Mammals only’ treatment − indicated by the 95% CI for *t*m not overlapping with 1.0 (Fig. 2).

Comparisons among the pollination treatments indicated that the pool of seedlings resulting from pollination solely by NFMs were characterized by slightly lower outcrossing rates (*t*m) and higher correlation of paternity (*r*p) relative to seedlings resulting from pollination by birds and insects, with the 95% CI for the group differences not overlapping zero (Fig. 2). There was no evidence of differences between the ‘Insects only’ pollination treatment and any other group (Fig. 2). Likewise, there was no evidence of difference between ‘Pollen supplementation’ treatment and ‘Open’ control.

### Seedling vigour and survival

**Fig. 3:**
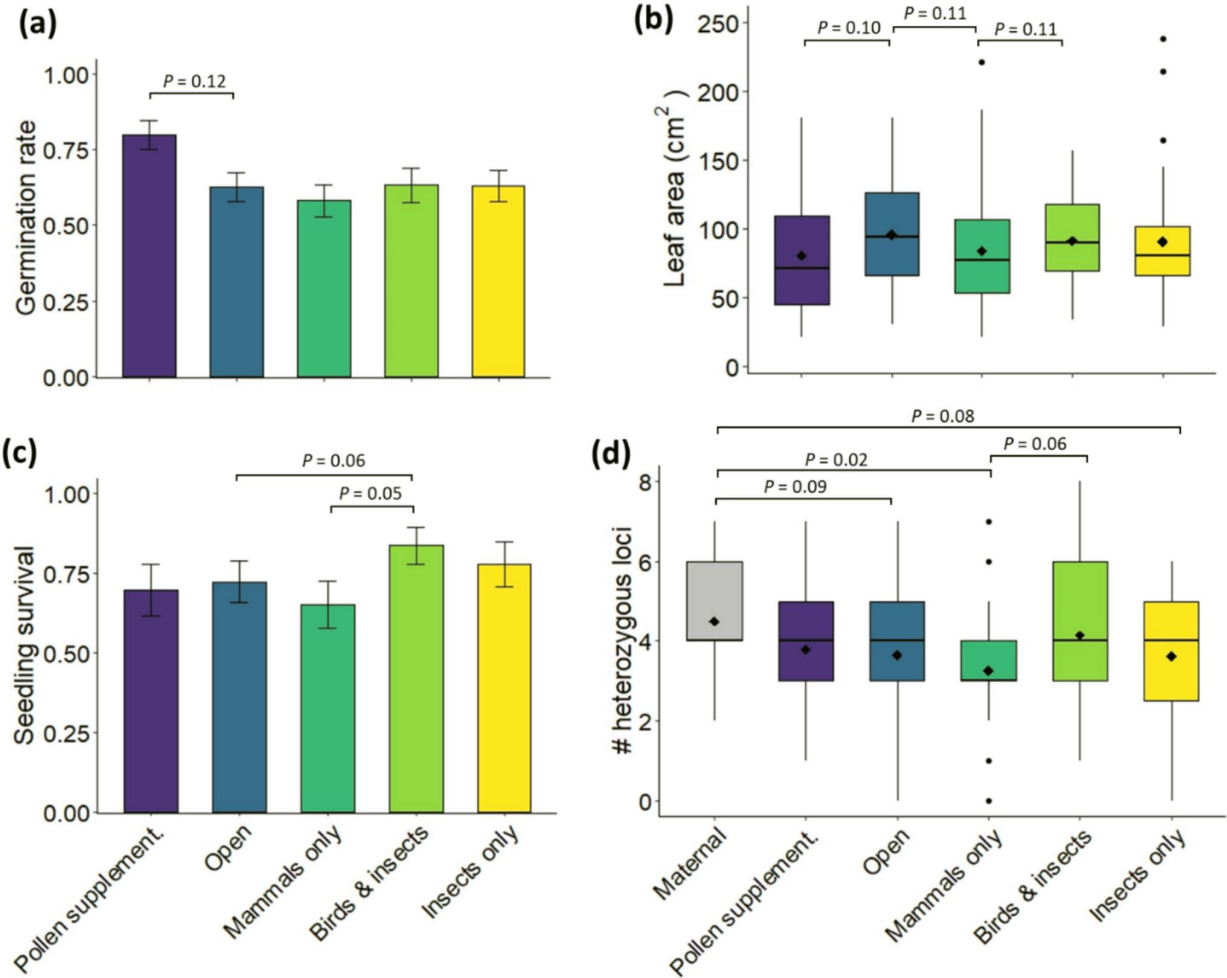
Comparison of: **a** seed germination rates, **b** seedling growth in the first 12 months, **c** seedling survival at 15 months (three months after transplanting to the field), and **d** individual heterozygosity (*H*i) among the maternal plants and experimental seedlings. *P*-values for pairwise comparisons between pollination treatments based on GLMMs as indicated, otherwise all *P*-values > 0.2 (omitted from the plot for clarity)

There was little variation among the pollination treatments in seed germination rates and seedling growth under shadehouse conditions for the first 12 months (Fig. 3a, b), with little to no evidence of difference for nearly all pairwise comparisons among groups of seedlings (GLMM, *P*-values > 0.2), except as indicated in Fig. 3. However, at 15 months (3 months after transplanting to the field site) the seedlings in the ‘Birds & insects’ treatment were more likely to survive than those in the ‘Mammals only’ treatment and ‘Open’ control, albeit the evidence for these differences was weak (GLMM, *P*-values 0.05–0.07; Fig. 3c).

### Individual heterozygosity

Comparison of individual heterozygosity (*H*i) indicated that the seedlings in the ‘Open-pollinated’ control were slightly less heterozygous than the maternal plants, with weak evidence of difference (GLMM, *P* = 0.09; Fig. 3d). However, while there was no evidence of difference in *H*i between the maternal plants and the seedlings in the ‘Birds & insects’ treatment (GLMM, *P* = 0.50; Fig. 3d), the seedlings in the ‘Mammals only’ treatment were less heterozygous than those in the ‘Birds & insects’ treatment, with weak evidence of difference (GLMM, *P* = 0.06; Fig. 3d). The seedlings in the ‘Mammals only’ treatment were also less heterozygous than the maternal plants, with moderate evidence of difference (GLMM, *P* = 0.02; Fig. 3d).

### Test of heterozygosity–fitness correlation

There was no evidence of correlation between seedlings’ individual heterozygosity (*H*i) and growth after 12 months under shadehouse (GLMM, *P* = 0.99; Table 3). However, there was moderate evidence of positive correlation between the seedlings’ *H*i and the probability of survival to three months after transplanting to the field (GLMM, *P* = 0.02; Table 3).

**Table 3:** Results of heterozygosity fitness correlation tests assessing the association between seedling individual heterozygosity (*H*i) and growth (projected green leaf area after 12 months under shadehouse conditions) and probability of survival at 15 months (3 months after planting in the field). Maternal plant was set as a random effect in the models (*N* = 22)

Response (model)	Predictor	*N* seedlings	Estimate	± SE	*P*
Seedling leaf area at 12 months (Gaussian)	*H*i	204	0.0245	1.67	0.99
Seedlings survival at 15 months (binomial)	*H*i	199	0.252	0.105	0.02

## Discussion

This study compared the genetic component of pollinator effectiveness among three different pollinator functional groups: birds, non-flying mammals (NFMs), and insects, in a shrub with floral traits suggesting adaptation to a mixed vertebrate pollination system. Our results show that pollination by NFMs was associated with slightly lower seedling heterozygosity, elevated rates of biparental inbreeding (*t*m < 1, despite evidence of obligate outcrossing) and correlation of paternity (*r*p), compared to pollination by birds and insects, but not insects alone. These results are consistent with the expectation that pollination by birds promotes wider outcrossing and paternal diversity, although these effects were subtle. Contrary to expectation, across all comparisons undertaken, we found no evidence that insects were less effective pollinators than the birds. Because it was not possible to selectively exclude insects while allowing birds to access the flowers, this result could be attributed to abundant visitation of the flowers by *A. mellifera*, which may have obscured the contribution of birds (Wawrzyczek et al. 2024a). Assuming that *B. catoglypta* is like other banksias studied, where nearest neighbours tend to be close relatives (Krauss et al. 2009; Ritchie and Krauss 2012), our results suggests that NFMs may disperse pollen over shorter distances relative to flying pollinators.

The results of GenAlEx suggest that exclusion of NFMs had the overall effect of increasing the genetic diversity of the seedling cohort (i.e. comparing ‘Birds & insects’ and ‘Mammals only’ treatment to ‘Open’ control). Further, this effect can be attributed largely to pollination by birds, because observed and expected heterozygosity (*H*o and *H*e) were considerably lower in the ‘Insects only’ treatment, compared to ‘Birds & insects’. The comparisons of mating system parameters (*t*m, *r*p) and individual heterozygosity (*H*i) could suggest a similar trend, as these parameters also tended to be lower in the ‘Insects only’ treatment compared to ‘Birds and insects’. However, our experiment provided no evidence of difference. Similarly, we found no evidence of difference for any of the parameters between ‘Insects only’ and ‘Mammals only’ treatments.

Biparental inbreeding, which was associated with pollination by NFMs, translated to slightly lower individual heterozygosity (*H*i) of the seedlings in the ‘Mammals only’ treatment, compared to those in the ‘Birds & insects’ treatment and maternal plants. We found no evidence that this reduction of *H*i influenced seedling growth under shadehouse conditions. However, the seedlings resulting from pollination solely by NFMs were slightly less likely to survive after transplanting to the field, compared to seedlings resulting from pollination by birds and insects. This result is consistent with the expectation of inbreeding depression in the (biparentally) inbred seedling cohort resulting from pollination by NFMs (Charlesworth and Willis 2009; Roberts et al. 2014), which is supported by the positive correlation between seedling survival and *H*i. These effects, however, were small—presumably because severe inbreeding, which could result from self-pollination, was prevented either through genetic self-incompatibility (Fuss and Sedgley 1991) or early acting inbreeding depression (Husband and Schemske 1996; Heliyanto et al. 2005). This, in itself, is an interesting result, suggesting that in obligately outcrossing species such as *B. catoglypta*, the differences in foraging behaviour among pollinator functional groups can have little consequence for plant mating. Given that the seedling survival was positively correlated with *H*i, inbreeding depression likely removes the least heterozygous seedlings before they reach maturity. This should prevent the genetic consequences of biparental inbreeding from being carried over to the next generation—an interpretation that is consistent with *H*i of the seedlings pollinated by birds and insects (with NFMs excluded) being equivalent to maternal plants.

Overall, the mating system of *B. catoglypta* was comparable to other banksias for which mating system data are available (Table 4). The comparison with other species is particularly useful because it provides a frame of reference for the magnitude of the differences in *t*m and *r*p associated with the exclusion of either NFMs or flying pollinators from the inflorescences of *B. catoglypta.* For example, in several cases when multiple populations of obligately outcrossing banksias were studied, the variation in *r*p among the populations of these species exceeded the difference between the ‘Mammals only’ and ‘Bird & insects’ treatments in our study (Table 4; see also a wider comparison in Kestel et al. 2021 and Coates et al. 2007). Admittedly though, the studies listed in Table 4 compared small and/or fragmented populations, so this comparison should be treated with caution.

**Table 4:** Comparison of the mating system parameters of *B. catoglypta* and other banksias from the same region where mating system was assessed using MLTR and either microsatellite or allozyme markers. *t*m = outcrossing rate; *r*p = correlated paternity

	*t*m	*r*p	References
*Banksia catoglypta*:			
Open-pollinated	0.92	0.09	This study
‘Mammals only’ –‘Birds & insects’	0.87–0.97	0.05–0.12	
*Banksia menziesii*	0.99–1.2	0.08–0.16	Ritchie et al. (2019)
*Banksia nivea*	0.94–0.96	0.05–0.19	Thavornkanlapachai et al. (2018)
*Banksia sphaerocarpa*	0.86–0.98	0.14–0.30	Llorens et al. (2012)
*Banksia cuneata*	0.62–0.94	0.07–0.16	Coates et al. (2007)
*Banksia oligantha*	0.85–1.0	0.03–0.10	Coates et al. (2007)

Between-study comparisons of *t*m are more difficult because this parameter depends on the power of the markers used (number and polymorphism), meaning that in most cases *t*m underestimates actual outcrossing rates (Ritland 2002). This appeared to be the case in our study, where due to low marker polymorphism some apparent selfing was indicated despite evidence that *B. catoglypta* is obligately outcrossing (Wawrzyczek et al. 2024a). Moreover, between- study comparisons of tm, should always consider the possibility of differences in the breeding system. Nonetheless, in other banksias the values of *t*m can be as low or lower than in our ‘Mammals only’ treatment, despite (presumably) birds also contributing to pollination (Table 4). These comparisons highlight the importance of considering population size, as small, isolated remnants can be prone to biparental inbreeding and correlated paternity, but this is not necessarily related to differences in pollinator community (Coates et al. 2007; Llorens et al. 2012).

The effects of *A. mellifera* on pollination of native flora is an important issue because these insects are super-abundant in southern Australia and visit a wide range of plant species (Paton 1993; Valido et al. 2019; Prendergast et al. 2022). The floral visitation by *A. mellifera* can disrupt mating of plants adapted to vertebrate pollination (Paton 2000) by removing nectar while contributing little to pollination (Ayre et al. 2020), which in some cases may drastically reduce plant fitness (Gross and MacKay 1998; Celebrezze and Paton 2004; Diller et al. 2022). *Banksia catoglypta* flowers in Austral winter when few native flower-visiting insects are active within its geographic range. Accordingly, *A. mellifera *was the only invertebrate pollinator frequently visiting the flowers (Wawrzyczek et al. 2024a). *A. mellifera* was introduced to Western Australia in 1846 and subsequently established feral colonies in much of the state (Wills et al. 1990). Locally, it is very abundant (Wawrzyczek et al. 2024a) and likely removes very large quantities of pollen and nectar from native plants. However, our results indicate that *B. catoglypta* may benefit from visitation by *A. mellifera* through increased pollen transfer (Wawrzyczek et al. 2024a) without negative consequences in terms of mating system parameters and vigour of the resulting offspring.

Very few previous studies have compared mating system parameters of plants pollinated by vertebrates and insects following a selective pollinator exclusion experiment, limiting our ability to make comparisons with the published literature. While some past studies suggest that *A. mellifera* may contribute less than birds to outcross pollination of self-compatible plants based on reduction of outcrossing rates following exclusion of birds (England et al. 2001; Schmidt-Adam et al. 2009; Bezemer et al. 2019), the results vary widely between species (e.g., Duffy et al. 2020, found no effect) and even between different populations of the same species (England et al. 2001; Bezemer et al. 2019). Thus, the effects of pollination by *A. mellifera* likely depend on some additional unrecognized factors, which could be idiosyncratic. Moreover, the results of these studies are difficult to interpret because it is generally not possible to selectively exclude insects while allowing birds to access the flowers (although see Diller et al. 2022). Studies (including ours) can demonstrate that *A. mellifera* contribute less than birds to outcross mating of plants they visit if there is a significant difference between ‘Birds & insects’ (or open-pollinated control, if NFMs are not involved) and ‘Insects only’ treatments. However, without a ‘Birds only’ treatment it is not possible to distinguish between the situation when (i) birds and insects are equally effective pollinators, and (ii) birds contribute more to outcrossed mating, but their contribution is ‘swamped’ due to more frequent visitation by *A. mellifera* (England et al. 2001). Our study is not free of this problem and therefore the results need to be interpreted with caution. However, our study is the first to conclusively demonstrate that *A. mellifera* can be at least as effective as NFMs as pollinators of an obligately outcrossing plant.

Looking more broadly beyond studies involving *Apis*, in self-compatible and facultatively autogamous *Protea caffra* from South Africa, exclusion of vertebrate pollinators had no effect on outcrossing rates (*t*m = 0.65 compared to 0.59 for open-pollinated plants, with no evidence of difference; Steenhuisen et al. 2012). This species is pollinated by birds and flower chafer beetles (Cetoniinae) which, when flying between plants, tended to move well beyond the nearest neighbouring plants, thus likely promoting outcross pollination (Steenhuisen et al. 2012). Thus, similar to the differences in foraging behaviour that have been documented for birds (e.g., relating to size and aggression, McFarland 1986; or between traplining and territorial hummingbirds, Torres-Vanegas et al. 2019) there likely also are wide differences among groups of pollinating insects. These differences are likely be more important for plant mating than the crude distinction of birds vs. insects.

The seedlings of *B. catoglypta* resulting from pollination solely by NFMs were least likely to survive, with evidence of biparental inbreeding leading to reduced heterozygosity and inbreeding depression. Moreover, the seedlings in ‘Open-pollinated’ control were less likely to survive than those in the ‘Birds & insects’ pollination treatment. Given that selective exclusion of NFMs from *B. catoglypta* did not lead to reduced fruit set in the year of the study (Wawrzyczek et al. 2024a), this unexpected result could suggest that, in the presence of other pollinators, floral visitation by NFMs can be superfluous and perhaps detrimental (since excluding NFMs improved seedlings’ survival). One interpretation of this result is that NFMs are interlopers to a plant that is primarily bird-pollinated, whereby visitation by NFMs contributes little to pollination and leads to a minor reduction in seed quality. This interpretation is difficult to evaluate because of the high frequency of visitation by the introduced *A. mellifera*, which could not be selectively excluded while allowing birds to access the flowers. However, the lower effectiveness of NFMs relative to other vectors is at odds with the possibility that the low, concealed flowers, and strong floral scent of *B. catoglypta* are traits that evolved under selection to favour pollination by NFMs—suggested by the presence of these traits in some primarily NFM-pollinated plants from South Africa (e.g., Biccard and Midgley 2009; Kühn et al. 2017) and other banksias where NFMs are frequent floral visitors (Wawrzyczek et al. 2024b). An alternative interpretation is that without pollination by introduced *A. mellifera*, fruit set of *B. catoglypta* would be pollen limited (at least in some years) if only birds were visiting the flowers, and therefore additional pollination by NFMs would have increased overall fruit set.

The suggestion that in the absence of *A. mellifera* fruit set of *B. catoglypta* would be pollen limited is supported by our camera trapping surveys of vertebrate visitation to the flowers in 2022, when *B. catoglypta* flowered particularly intensively (Wawrzyczek et al. 2024a). These surveys revealed an overall low frequency of visitation to individual inflorescences, with some of the flowering branches that were monitored receiving only one or no visits by vertebrate pollinators throughout anthesis (Wawrzyczek et al. 2024a). Moreover, in three other *Banksia *species in the same region where *A. mellifera* was either a less frequent or less effective pollinator, exclusion of NFMs was associated with substantial reduction of fruit set compared to open-pollinated controls (Wawrzyczek et al. 2024b). Thus, historically, there may have been an advantage to attracting NFMs as secondary pollinators that increased fruit set, despite the slight negative fitness consequences for individual seedlings.

In conclusion, we showed that in *B. catoglypta,* pollination by birds and *A. mellifera* led to near-equivalent outcrossing, paternal diversity, seedling heterozygosity and vigour. In comparison, pollination solely by NFMs (honey possums and mice) was associated with biparental inbreeding and correlated paternity, with resulting seedlings characterized by lower heterozygosity and vigour. However, the differences were surprisingly slight and likely of little consequence for genetic diversity of the *B. catoglypta* population, as inbreeding depression should select against low heterozygosity seedlings in early life-stages. Although the floral traits of *B. catoglypta* may suggest adaptation to pollination by NFMs, our results, together with those of Wawrzyczek et al. (2024a), indicate that NFMs are less effective pollinators than birds and insects, and that currently floral visitation by NFMs may even be slightly disadvantageous. However, prior to introduction of *A. mellifera*, fruit set of *B. catoglypta* may have been frequently pollen-limited, in which case pollination by NFMs would be overall advantageous through provision of additional pollination service alongside honeyeaters.

Supplementary information Supplementary material 1: Primer sequences and details of PCR protocol.

## Supplementary Information

Below is the link to the electronic supplementary material.

Supplementary file1 (DOCX 40 KB)

## Data Availability

Microsatellite genotypes, cotyledon emergence, seedling growth and survival data are made available via GitHub:  https://github.com/stanwawrzyczek/Banksia_genetic_component
