# Supplementary material for: Experimental comparison of the genetic component of pollinator effectiveness in a shrub pollinated by birds, non-flying mammals and European honeybees
Source: Oecologia. 2025 Jun 6;207(7):101. doi: 10.1007/s00442-025-05736-x (PMC12144075; doi:10.1007/s00442-025-05736-x)
Supplement: Supplementary file 1 — Supplementary file1 (DOCX 40 KB) [file 442_2025_5736_MOESM1_ESM.docx]

**Supplementary Material 1**. Microsatellite primers and details of PCR protocol.

Experimental comparison of the genetic component of pollinator effectiveness in an obligately outcrossing shrub pollinated birds, non-flying mammals and European honeybees.

STANISLAW K. WAWRZYCZEK^1*^ ; SIEGFRIED L. KRAUSS^2,3^; SUSAN E. HOEBEE^1^ and RYAN D. PHILLIPS^1,2,4,5^

^1^ *Department of Environment and Genetics, La Trobe University, Melbourne, VIC 3086, Australia*

^2^ *Kings Park Science, Department of Biodiversity, Conservation and Attractions, Fraser Avenue, Kings Park, WA 6005, Australia*

^3^ *School of Biological Sciences, The University of Western Australia, Crawley, WA 6009 Australia.*

^4^ *Ecology and Evolution, Research School of Biology, The Australian National University, Canberra, ACT 2601, Australia*

*^5^ Royal Botanic Gardens Victoria, Melbourne, VIC 3004*

* author for correspondence

| **Table S1.** Microsatellite primer sequences provided by Australian Genome Research Facility (excluding M13 primer sequences). Estimated Tm of all primers was 60 °C ± <1 °C. In bold primers | | | | |
| --- | --- | --- | --- | --- |
| Locus | Fwd. primer sequence | Rev. primer sequence | Motif | Target region length (bp) |
| **Bca01** | ATTGCACTTGGGTCGTTTGC | GAGTCATACGGAGCCAGGTG | AAC | 21 |
| **Bca02** | TTCAGCCGGAGTTCACTCAC | AACGCTACTCCTTCAGCTCG | AAG | 15 |
| **Bca03** | TCCCAACTTATCCAACCGCG | GATGCCGATCTGGTGTAGGG | AC | 12 |
| **Bca04** | TTGGCCAATTCCGTTCATGC | CGATTTGGCGTGTGGAAGTG | AAC | 18 |
| **Bca05** | AAGTGCTGTGCAGAGTCCAA | AACTCAACAGGGAGGTTGGC | AAC | 24 |
| **Bca06** | CCCGAGAAGCTCTTCCTTGG | CGACACTGGTACTGCTAGGG | AAG | 18 |
| **Bca07** | AGTTGCAGGCACCATGAAGA | TCCACGGCTTGGTAAGAGTG | AC | 14 |
| **Bca08** | CTCTCGGTTGTGCCCTTCTC | AGGAGAGCTGCAATATGGGC | AAG | 15 |
| **Bca09** | GGAAGACTCCTCAAACGGCA | TTGCACTGAGAATCTGCCCA | AC | 14 |
| **Bca10** | GGACAAGGATCCGTGTAGCC | TGCTGCTTGGGCTATACCTG | AAC | 30 |
| Bca11 | GCACCGGAGTTCTTAAGGCT | GAATGCAAACCCAGCAGCAA | AAC | 15 |
| Bca12 | GGTATGTAGCAGGACTGGCC | GAGGCTGGAGCAGTTGAAGT | AAC | 15 |
| Bca13 | CGCCAACCCACAAATTTGGT | GGATCCATGTAGCCGACTCC | AAC | 15 |
| Bca14 | GCCTTGATTGCTCGGGATCT | TCTCAGTGAAAGCAGCCTCG | AAG | 15 |
| Bca15 | CATATGGGTGGGTCAGGGTG | TCGAGTTGGGCAGCTGAATT | AAG | 15 |
| Bca16 | CTTTCTCTGCCACCGGGAAA | CCTCCTGAACTCCTCTGGGT | AAG | 24 |
| Bca17 | TCAGTTCCAAGCAGAAGGGC | GTGGTGGCAGTATGGGATGT | AAG | 18 |
| Bca18 | CCTCGCTACTTTGGCTTTGC | AGTAAGCAAGAGTGTGCCGT | AAG | 33 |
| Bca19 | ACTTCAAACCACAGCCCTGT | TACAAGTTGTCCCACCACCG | AC | 10 |
| Bca20 | TGTCGTGACAGAGTTGGCTG | CCGACTGCATTGCTTCTTGG | AC | 10 |

| **Table S2**. Tail sequences attached to the forward primer (Blacket *et al.*, 2012) | |
| --- | --- |
| **Dye** | **Sequence** |
| 6-FAM | GCCTCCCTCGCGCCA |
| VIC | GCCTTGCCAGCCCGC |
| NED | CAGGACCAGGCTACCGTG |
| PET | CGGAGAGCCGAGAGGTG |

| **Table S3** Multiplex PCR design. | | | | | | | |
| --- | --- | --- | --- | --- | --- | --- | --- |
|  | **Primer pair** | **Dye** | **Repeat (bp)** | **allele size range**  **(min-max, bp)** | | **N alleles detected** |  |
| **Multiplex A** | Bca01 | VIC | 3 | 174 | 183 | 3 |  |
|  | Bca02 | NED | 3 | 181 | 184 | 2 |  |
|  | Bca03^a^ | PET | 2 | 263 | 304 | 1 |  |
|  | Bca04 | PET | 3 | 270 | 310 | 3 |  |
|  | Bca05 | NED | 3 | 268 | 277 | 4 |  |
|  | Bca06^b^ | VIC | 3 | 297 | 315 | 6 |  |
| **Multiplex B** | Bca07 | 6-FAM | 2 | 149 | 153 | 4 |  |
|  | Bca08 | VIC | 3 | 200 | 206 | 2 |  |
|  | Bca09 | NED | 2 | 215 | 223 | 5 |  |
|  | Bca10 | 6-FAM | 3 | 274 | 292 | 3 |  |

^a^ Locus discarded as apparently monomorphic

^b^ locus discarded due to apparent polysomy (amplifying up to 4 peaks)

**Table S4**. PCR conditions used with Type-it Microsatellites Kit (Qiagen 2016).

| **Step** | **Temp (˚C)** | **Duration (min)** | **Repeat** |
| --- | --- | --- | --- |
| initial | 95 | 5 | x1 |
| denature | 95 | 0.5 | x25 |
| anneal | 57 | 1.5 | x25 |
| extend | 72 | 0.5 | x25 |
| final | 60 | 30 | x1 |
|  |  |  |  |

| **Table S5.** Multiplex A (15µL reactions) | | | |
| --- | --- | --- | --- |
|  |  | **Final conc. (µM)** | **vol x1 (µL)** |
| Type-it Master Mix (x2) |  | x1 | 7.5 |
| F primers | **Bca01F** | 0.1 | 0.15 |
|  | **Bca02F** | 0.1 | 0.15 |
|  | **Bca03F** | 0.1 | 0.15 |
|  | **Bca04F** | 0.1 | 0.15 |
|  | **Bca05F** | 0.1 | 0.15 |
|  | **Bca06F** | 0.1 | 0.15 |
| Labelled universal primers | FAM | 0 | 0 |
|  | VIC | 0.3 | 0.45 |
|  | NED | 0.3 | 0.45 |
|  | PET | 0.3 | 0.45 |
| R primers | Bca01R | 0.25 | 0.375 |
|  | Bca02R | 0.25 | 0.375 |
|  | Bca03R | 0.25 | 0.375 |
|  | Bca04R | 0.25 | 0.375 |
|  | Bca05R | 0.25 | 0.375 |
|  | Bca06R | 0.25 | 0.375 |
| H_2_O |  |  | 1 |
| DNA template (20-100ng/µL) |  |  | 1 |

**Table S6.** Multiplex B (15µL reactions)

|  |  | **Final conc. (µM)** | **vol x1 (µL)** |
| --- | --- | --- | --- |
| Type-it Master Mix (x2) |  | x1 | 7.5 |
| F primers | **Bca07** | 0.1 | 0.15 |
|  | **Bca08** | 0.1 | 0.15 |
|  | **Bca09** | 0.1 | 0.15 |
|  | **Bca10** | 0.1 | 0.15 |
|  |  |  |  |
|  |  |  |  |
| Labelled universal primers | FAM | 0.3 | 0.45 |
|  | VIC | 0.16 | 0.24 |
|  | NED | 0.16 | 0.24 |
|  | PET | 0 | 0 |
| R primers | Bca01R | 0.25 | 0.375 |
|  | Bca02R | 0.25 | 0.375 |
|  | Bca03R | 0.25 | 0.375 |
|  | Bca04R | 0.25 | 0.375 |
|  | Bca05R | 0.25 | 0.375 |
|  | Bca06R | 0.25 | 0.375 |
| Water |  |  | 2.72 |
| DNA template (20-100ng/µL) |  |  | 1 |
